# Anaesthesiologist-intensivist phycisians at the core of the management of critically ill COVID-19 patients in Africa: persistent challenges, some resolved dilemma and future perspective

**DOI:** 10.11604/pamj.supp.2020.37.1.25234

**Published:** 2020-12-11

**Authors:** Junnette Arlette Mbengono Metogo, Joel Noutakdie Tochie, Paul Owono Etoundi, Roddy Stephan Bengono Bengono, Raymond Ndikontar, Jacqueline Ze Minkande

**Affiliations:** 1 Department of Surgery and Sub-Specialties, Faculty of Medicine and Biomedical Sciences, University of Yaoundé 1, Yaoundé, Cameroon,; 2 Department of Anaesthesiology and Critical Care Medicine, Douala General Hospital, Douala Cameroon,; 3 Department of Anaesthesiology and Critical Care Medicine, Yaounde Gynaeco-Obstetric and Pediatric Hospital, Yaoundé, Cameroon,; 4 Department of Emergency, Anaesthesiology and Critical Care Medicine, Yaoundé Central Hospital, Yaoundé, Cameroon

**Keywords:** COVID-19, severe form, intensivist, Africa, management, challenges, solutions, perspectives

## Abstract

Unlike developed countries which have purely intensivists also called critical care physicians or intensive care physicians to manage critically ill patients like those with severe forms of COVID-19, the practice of critical care medicine in Africa is coined to anaesthesiology. Hence, anaesthesiologist-intensivist physicians are the medical specialists taking care of critically ill COVID-19 patients in Africa. Likewise, unlike intensive care units (ICUs) in high income countries, those in most African countries face the challenge of a lack of emergency drugs and resuscitation equipment, limited health infrastructure and understaffed and underfunded health care systems. The COVID-19 pandemic is an unprecedented one faced by intensivists in high-income countries and anaesthesiologist-intensivist phycisians in Africa. Infected patients with severe forms of the disease like those having grave COVID-19 complications like massive pulmonary embolism, severe cardiac arrhythmias, cardiogenic shock, septic shock, acute kidney injury or acute respiratory distress syndrome require ICU admission for better management. Both intensivists or anaesthesiologist-intensivist physicians have the peculiarity of securing the airways of critically COVID-19 patients and providing respiratory support with mechanical ventilation after laryngoscopy and endotracheal intubation when needed. In so doing, they can easily be infected from respiratory droplets or aerosols expired by the COVID-19 patients. Hence, in Africa, anaesthesiologist-intensivist phycisians have a higher risk of contracting COVID-19 compared to other health professionals. It's worth to mention that the COVID-19 pandemic struck African anaesthesiologist-intensivist phycisians and ICUs when there were neither prepared skillfully or lacked the required ICU capacity to meet the demands of thousands of severe COVID-19 African patients. These further weakened the already strained health systems in Africa. It required a lot of creativity, engineering skills and courage for these ill prepared African anaesthesiologist-intensivist physicians to provide care to these critically ill patients and improve their outcomes as the pandemic progressed. However, despites the numerous efforts made in African anaesthesiologist-Intensivist phycisians to care for critically ill COVID-19 patients, the pandemic is spreading at a rapid rate across Africa. There is an urgent need for African health authorities to anticipate on how to scale up the future high ICU capacity needs and limited ICU workforce, infrastructure and equipment to manage severe forms of COVID-19 in future. It cannot be overemphasized that these severe forms of COVID-19 are potentially fatal and are a major contributor to the death toll of the COVID-19 pandemic.

## Essay

A recent pandemic of a highly contagious and potentially fatal respiratory infection called coronavirus disease (COVID-19) emerged in December 2019 in China. COVID-19 is caused by a viral pathogen called “severe acute respiratory syndrome-coronavirus 2” (SARS-CoV-2) [1]. Within three months of its outbreak, this life-threatening communicable disease spread around the world so rapidly than no unprecedented infection, contaminating over three million people of which 274,985 died worldwide [2]. Fatalities are often due to severe forms of the disease which are potentially lethal especially if untreated or inadequately managed [3]. These severe forms of the disease require intensive care management which is quite difficult in Africa where the anaesthesiologist-intensivist physicians´ (called the critical care physicians or the intensive care physicians in developed countries) workforce is low and intensive care units have limited equipments to resuscitate these patients with severe forms of COVID-19 [4].

**Persistent challenges confronted by anaesthesiologist-intensivist phycisians managing severe forms of COVID-19 in Africa:** the African continent is reputable as one of the most financially poor regions on the globe, with a double global health burden stemming from a the constant threat of infectious diseases and a rising burden of non-communicable diseases due to the epidemiological transition of diseases [1]. To the high burden of communicable diseases such as HIV/AIDS confronted by Africa, COVID-19 abruptly added as a de novo very infectious respiratory tract infections in March 2020 as a global health crisis called a pandemic. COVID-19 is currently ranked as the primary priority of the global burden of disease (GBD) [2]. COVID-19 can manifest as mild and severe forms. The former includes benign systemic symptom (rhinorrhea, fever, gastrointestinal upset, fatigue, headaches) which can be managed and followed-up at home or may neccessitate a short length of hospital stay for management. On the other hand, severe COVID-19 presents with emergencies such massive pulmonary embolism, acute repiratory distress syndrome (ARDS), myocardial infarction, heart failure, septic shock, and acute kidney injury which warrant immediate ICU admission for adequate intensive care to safe the live of these patients with severe COVID-19. The global prevalence of severe COVID-19 is estimated between 15 - 20% [3,4], quite worrisome to be labeled as trivial. Due to its unprecedented occurrence, the COVID-19 pandemic struck all anaesthesiologist-intensivist physicians and ICUs in Africa like a tornado. All medical specialists in Africa were not prepared to manage patients with a disease they had never heard nor been confronted with. Nonetheless, they had to procure optimal case management to severe forms of COVID-19. Compared with Western countries, the emergence of COVID-19 was more terrific due to a prevailing poverty, lack of emergency drugs and resuscitation equipment, few health infrastructure and understaffed and underfunded healthcare systems [3,4] which challenged the various national, local and United Nations system responses to the pandemic [5]. These culminated to further strain the already weak healthcare systems in Africa. Overall, 769 confirmed African cases of critically ill COVID-19 and case fatality rate of 0.5% were recorded by March 20, 2020, compared with 770 956 severe COVID-19 African cases and case fatality rate of 2.3% by July 27, 2020 [2,6].

The average ICU bed capacity in most African countries is less than 24 ICU beds (if not none) [6]. For instance, Kenya has a total of 400 ICU, Nigeria about 120 ICU beds and Cameroon has 601 ICU beds [6]. WHO reports an estimate five ICU beds per one million person in Africa compared with 4000 beds per one inhabitants in Europe [4]. More reliable national African estimates depict none to 17 ICU beds per 100,000 population in Egyptians, 6 beds per 100,000 persons in Seychelles and 9 beds per 100,000 South Africans [7]. The average ICU bed capacity in most African countries is less than 24 ICU beds (if not none) Uganda in particular is has been repeatedly noted to have a significant ICU bed capacity and Ugandans have limited access to intensive care services [8]. It is extrapolated that more than 50% of the global population will be infected by COVID-19 over the next two years and the burden of this infection will disproportionately affect Africans, the limited Africa´s ICU workforce, and ICU´s capacity to provide proper healthcare. As the pandemic progresses, although its pathophysiology, diagnosis, and provisionally validated treatment protocols (while waiting for the discovery of a definite treatment and vaccine) are been understood by African anaesthesiologist-intensivist physicians, patients who become critically ill of COVID-19 still require weeks of treatment in African ICUs and many undergo endotracheal intubated and mechanically ventilated to have a chance of survival [9]. In the same vein, Africa faces significant financial constraints to set up ICUs for the provision of critical care to those suffering from the most severe cases of COVID-19 [1].

Indeed, the burden of critical illness in most African countries is overwhelming and there is an unmatched number of ICU beds, ventilators, electrocardiograms, ultrasound machines, and defibrillators estimated at one per a million critically ill COVID-19 patients. Furthermore, the international market is saturated with demands of ICU equipment from high-income countries and international transportations of these ICU equipment are also a challenge due to the reduced cargo space recently implemented by airlines stopping their services to Africa [1]. Apart from the shortage of ICU beds in Africa, the burden of severe COVID-19 patients is further compounded by a drastic paucity of workforce from African anaesthesiologist-intensivist physicians, anesthetist-ICU nurses and respiratory physiotherapists for the optimal management of these critically ill patients. Anaesthesiology and critical care medicine is a unique, challenging, dynamic medical specialty in Africa requiring skilled personnel to adequately manage patients in the ICU. Unfortunately, these skilled labour remains a scarce invaluable asset in Africa [4]. Another barrier to the optimal care of severe COVID-19 cases in Africa is the shortage of personal protective equipment (PPE) and mechanical ventilators to provide respiratory support to patients with ARDS [6]. Several COVID-19 patients with ARDS in Africa have died due to lack or insufficient ventilators. According to WHO, less than 2000 ventilators are available in 41 African countries. For example, in Mali, there are only 56 ventilators for 19 million inhabitants [9]. A similar trend was observed in Cameroon on the 29^th^ March 2020, where a national survey reported a total of 73 mechanical ventilators for 23 million inhabitants and a case fatality rate of 60% among severe COVID-19 patients [10].

**Solutions adopted by anaesthesiologist-intensivist phycisians managing severe forms of COVID-19 in Africa:** the pandemic had major adverse repercussions on the African ICUs. It required a lot of creativity, engineering skills and bravery from the un-prepared African anaesthesiologist-intensivist physicians to provide care to these critically ill patients and improve patients´ outcomes as the pandemic progressed. Within few months of its outbreak the baseline ICU capacity had already been reached worldwide and particularly in Africa [9]. For instance, some creative initiatives undertaken in Africa was the conversion of several operating rooms, old hospitals and public spaces like schools, olympic stadiums and supermarkets into ICUs with subsequent assignment of intensivists to head and manage these annexed ICUs [9]. These measures slightly helped the African continent to be able to match the high ICUs demands of severe COVID-19 patients. However, the major challenge of these annexed ICUs has been a lack or paucity of mechanical ventilators to provide respiratory support to patients with severe COVID-19 when needed. Furthermore, critically ill COVID-19 patients require a long length of ICU stay, and once elective surgeries postponed during the pandemic to give preference to emergency surgeries progressively resume the ICU capacity in Africa would be more challenged [11]. However, as several African countries scramble to create ICU capacity through makeshift beds in theaters, old hospitals and public places, important questions will arise such as (a) will ICU services maintain an effective health workers-to-patient ratio? (b) Will a higher number of ICU bed capacity spread the resources thinner than before? (c) Will more ICU beds not predispose intensivists and ICU nurses to a burnout syndrome and increase their risk being infected by the viral pathogen causing COVID-19? [9].

**Future perspectives in the management of critically ill COVID-19 patients by African anaesthesiologist-intensivist phycisians from the Cameroonian case study:** though the number severe cases are increasing in some African countries like Cameroon, now considered the COVID-19 epicenter in Western and Central Africa [10], the severe forms of COVID-19 are now been better managed with lesser deaths thanks to the joint efforts of the United Nations International Children´s Education Fund (UNICEF), United Nations Population Fund (UNFPA), the World Health Organization (WHO) and the Cameroon Ministry of Public Health [12,13]. UNICEF limited the spread of COVID-19 to African anaesthesiologist-intensivist physicians and other medical specialists via a donation of PPE (5680 coverall protection, 6950 non-sterile gowns, 2450 N95 masks and 7850 surgical masks) to the Cameroonian Ministry of Public Health [12]. In response to the increasing trends in COVID-19 infected individuals, the high demand for PPE, and the low supply of PPE, the UNFPA donated an emergency assistance to the Cameroon National Health Emergency Operations Center [13]. The grant consisted of several packets of medical shoe covers, disposable gowns, examination and surgical gloves, masks, hand sanitizer gel and many others [13]. Likewise, at the outbreak of COVID-19, the Cameroon Ministry of Public Health in collaboration with epidemiologists and anaesthesiologist-intensivist physicians elaborated a treatment protocol for the treatment of mild and severe forms of COVID-19 nationwide [12]. Meanwhile, the Ministry of Public Health in Cameroon is constantly formulating measures for disease prevention, thereby, reducing the number of new severe cases [12]. Cameroon has also received support to fight against COVID-19 from several countries including Morocco which offered free of charge PPE worth hundreds of millions XAF in a bid to foster a sustainable mutually beneficial bilateral relations between the Kingdom of Morocco and the Republic of Cameroon [14]. Lastly, in an unpublished report, WHO authorities together with the Ministry of Public Health and the Cameroon Society of anesthesiologist, intensivists and emergency physicians organized two training session in June 2020 to capacitate Cameroonian anaesthesiologist-intensivist physicians on effective case management of COVID-19, especially patients with severe forms of the disease to reduce of their case fatality rate. Unfortunately, despite some of the above cited public interventions for primary prevention of COVID-19; therapeutic and health logistic resolutions taken by African Health authorities for secondary and tertiary prevention of COVID-19 in Africa, statistics show that the number of new infections, new severe forms and deaths are increasing daily [2].

To this effect, we recommend the following measures to mitigate the impact (number of new infected severe cases and deaths) of COVID-19 in Africa. Firstly, a reinforcement of Africans adherence to WHO's guidelines on preventive measures like regular hand washing, wearing of a face masks and physical distancing. Secondly, Africans should avoid unnecessary travelling and stay away from overcrowded areas especially if they have risk factors for contracting COVID-19 such as being aged over 60 years, having comorbidities such as obesity, cardiovascular diseases, diabetes, chronic respiratory disease or malignancies. Thirdly, there is a continuous need to train African anaesthesiologist-intensivist physicians in leadership and adequate management of severe forms of COVID-19 during this period of panic. Furthermore, although local regional anesthesia is the anaesthetic technique of choice for COVID-19 patients depending on the indication of surgery, intravenous anaesthesia is still indicated in the management of COVID-19 patients undergoing emergency surgical procedures [15]. Here, Opioid Free Anaesthesia (OFA) has the merit of a better anaesthetic technique compare to general anaesthesia mainly due to haemodynamic stability and the no postoperative respiratory depression compared with general anaesthesia [15]. Hence, OFA´s indication in COVID-19 patients undergoing emergency surgery should be considered by Anaesthesiologists for a better postoperative outcome of severe cases of COVID-19. Lastly, African anaesthesiologist-intensivist physicians should avoid as much as possible to relief severe acute or chronic pain using morphine or its synthetic derivatives in critically ill COVID-19 patients with respiratory compromise as these may worsen or precipitate respiratory distress in these patients.

## Conclusion

Although the brilliant initiatives of African anaesthesiologist-intensivist physicians confronted with this unprecedented pandemic is commendable, there is an urgent need for all health organizations (WHO, UN, CDC, African Ministries of Health) and all critical care physicians´ associations across the globe to brainstorm on how to improve the capacitating of critical care medicine in Africa with more skillful human resources (African anaesthesiologist-intensivist phycisians and Anaesthetist-ICU nurses), PPE, ICU beds, emergency drugs and ventilators geared at saving the lives of critically ill COVID-19 patients.
